# Flow-induced surface crystallization of granular particles in cylindrical confinement

**DOI:** 10.1038/s41598-021-92136-9

**Published:** 2021-06-24

**Authors:** Sheng Zhang, Ping Lin, Mengke Wang, Jiang-feng Wan, Yi Peng, Lei Yang, Meiying Hou

**Affiliations:** 1 Institute of Modern Physics, Chinese Academy of Sciences, Lanzhou, 730000 China; 2 University of Chinese Academy of Sciences, Beijing, 100049 China; 3 East China University of Technology, Nanchang, 330105 China; 4 Lanzhou University, Lanzhou, 730000 China; 5 Institute of Physics, Chinese Academy of Sciences, Beijing, 100190 China

**Keywords:** Self-assembly, Rheology

## Abstract

An interesting phenomenon that a layer of crystallized shell formed at the container wall during an orifice flow in a cylinder is observed experimentally and is investigated in DEM simulation. Different from shear or vibration driven granular crystallization, our simulation shows during the flow the shell layer is formed spontaneously from stagnant zone at the base and grows at a constant rate to the top with no external drive. Roughness of the shell surface is defined as a standard deviation of the surface height and its development is found to disobey existed growth models. The growth rate of the shell is found linearly proportional to the flow rate. This shell is static and served as a rough wall in an orifice flow with frictionless sidewall, which changes the flow profiles and its stress properties, and in turn guarantees a constant flow rate.

## Introduction

Self-assembly widely appears in nature, from ripples in sand, waves of the sea, to spirals on the shell of snails and bacterial snowflakes^1–4^. Ordering phenomena are also widely studied in driven disordered system^5–8^. As a non-equilibrium model system, agitated granular matter has often been used in the lab to study mechanisms behind these ordering phenomena. Particle alignment, related to ordering, rheology and entropy in disordered systems, has frequently been induced and investigated in shearing, twisting, shaking or inclined-flow granular media^7,9–11^. Different from these external agitations induced granular ordering, we report in this work observation of a layer of shell crystallized spontaneously at the container wall during an orifice flow without external agitation.

Flow of granular material from a hopper through an orifice, namely hopper flow here, is featured by its constant flow rate. The flow rate can be described by Beverloo’s scaling law quantitatively:

1 $$ W = C\rho \sqrt g \left( {D_{0}  - kd_{0} } \right)^{{2.5}}  $$

where ${D}_{0}$ denotes outlet size, $\rho$ is apparent density (the mass per unit volume in the container) of the granular material and $g$ is gravitational acceleration. $C$ and $k$ are empirical constants which depend on the grain and container properties, such as friction coefficients, particle shape or hopper angle^12–14^. The constancy of the flow rate may be related to either dynamical Janssen effect or existence of a ‘free fall arch’ region^15^ over the outlet. Continuum modeling of hopper flow by Staron et al. successfully reproduced the constant flow rate and pressure cavity by implementing a plastic rheology in Navier–Stokes’s solver^16^. These findings imply that when discharging from a hopper, granular material resembles other visco-plastic fluids with a shear stress changing from shear rate *γ*-independent in elastic limit to $\sim {\gamma }^{2}$ in rapid shear flow^17^ as described by Bagnold already in 1954^18^.

Several recent studies focused on frictional properties of the particles and the silo wall which are crucial in setting the flow profile and the flow rate. Experimental observations by X-ray tomography^19^ or electrical capacitance tomography^20^ showed, that increasing wall roughness leads to increasing thickness of the shear zone near the wall for sand. Similarly, DEM simulations by Gonzalez show that increasing wall roughness leads to a mass flow-funnel flow transition^21^. The flow rate from a silo was reported to systematically decrease with increasing the surface roughness of the particles^22^ or with increasing internal angle of friction of the granular material^23^. In DEM simulations by Vidyapati the flow rate decreased with increasing interparticle friction, but was insensitive to the wall friction^24^.

In this work we report a peculiar phenomenon observed in experiment and investigated in simulation, that during the drainage a crystallized granular shell develops next to the wall. Development of the shell gradually changes the flow profile inside the silo as it leads to a change in the friction at the boundary without changing the interparticle friction in the bulk. We explore the process leading to the formation of the shell and analyze whether it influences the flow rate. The development of crystalline ordering was reported in other (sheared or shaken) granular systems before^6,10,25–28^, where the volume fraction and particle–particle friction played an important role. The initial crystallized ‘nucleus’ appeared not only at the boundary but also in the central region^10,29^. Other examples, such as for inclined flow under gravity, ordering arises upon ordered base or driven by the side wall friction^11,30^. Our observation of the remaining crystallized granular shell also reminds for comparison with the slow motion of retention of viscous fluid on a vertical plate as investigated by Jeffreys in 1930^31^ and by Gutfinger and Tallmadge^32^ in non-Newtonian fluids. Compared to fluids, there are two major differences in the granular case: (1) the formation of a crystallized shell is a dynamical process grown upwards from bottom; (2) the crystallized shell in our case grows during the drainage and is still mechanically stable after drainage.

## Results and discussions

### Formation of the shell

**Figure 1:**
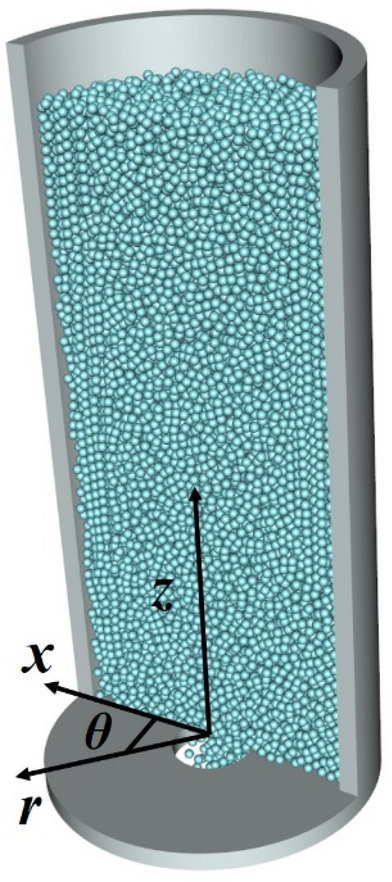
Simulation system with a cylindrical coordinates system. The origin is set at the center of the bottom.

Crystallized shells of particles next to the wall during the drainage are both observed in our experiments and simulations (shown in Video 1, Video 2, and Video 3). Numerical simulation investigates the dynamics of the shell formation and its influencing factors in a cylindrical hopper with a flat bottom (see Fig. 1). We use *D* as the hopper diameter and *d* as the particle diameter while *d*_0_ as the averaged particle diameter in a particular simulation case. In order to describe the growth of crystallized shells, three types of particles are defined here as one gets closer to the wall: ***boundary particle***, if its radial coordinate is greater than *D*/2-*d*_0_, to ensure no particle locate between this particle and the wall; ***wall particle***, if the particle touches the sidewall (i.e., radial coordinate is equal to or greater than *D*/2-*d*_0_/2), and ***shell particle***, which is a wall particle touching six neighboring wall particles to form a nearly static crystallized shell. To define top surface of the shell, the boundary layer (all the particles with an *r*-coordinate larger than *D*/2-*d*_0_) is divided into several vertical columns. In each column the shell particles are recognized from bottom to top. The last shell particle in this column is defined as the shell particle *k* when there is no other shell particle located in the range (*z*_*k*_, *z*_*k*_ + 20*d*_0_), where *z*_*k*_ is the *z* coordinate of particle *k*.

**Figure 2:**
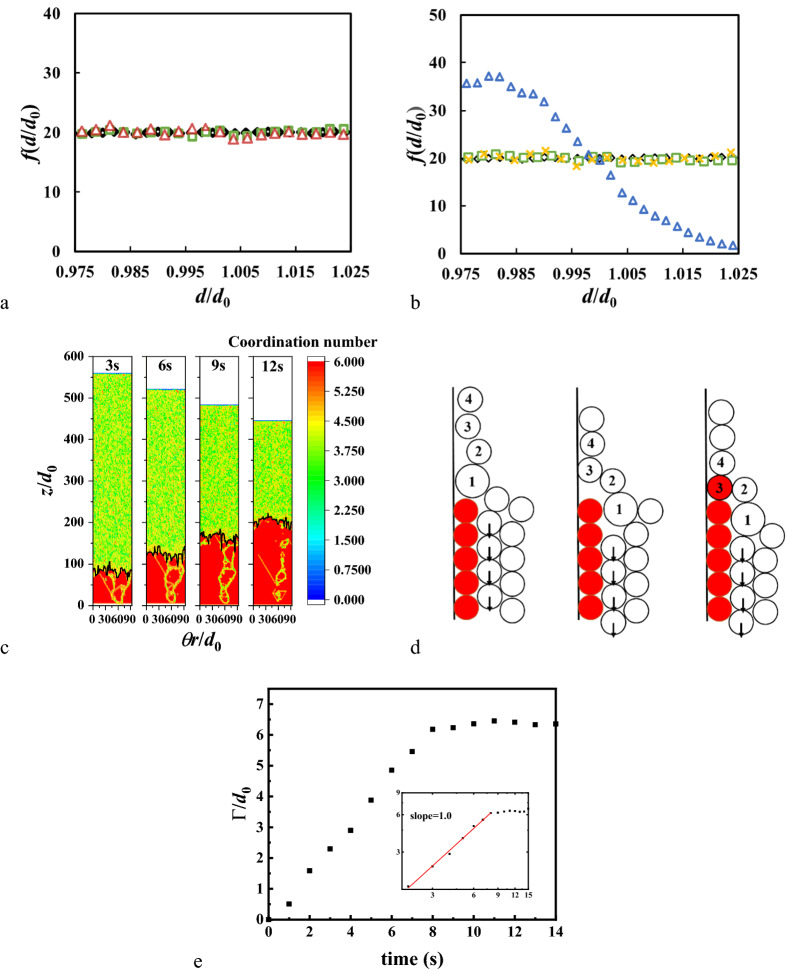
(**a**) Frequency distribution of initial radial coordination of shell particles. Size distribution of different particle groups with (**a**) *μ*_pp_ = 0.5; (**b**) *μ*_pp_ = 0.05. Diamond (black): all particles in the hopper. Square (green): particles at the boundary when the flow starts. Upper triangle (blue): the shell particles when the drainage is over. X (yellow): the boundary particles above the upper surface during the drainage. Upper triangle (red): the boundary particles when flow is stable. (**c**) Side area of the cylindrical hopper at different time (3, 6, 9, 12 s after the flow starts), showing the growth of the crystallized shell. The color denotes the coordination number of wall particles. The red particles are shell particles. Black lines denote the local surface of the shell. (**d**) Illustration of the shell growth. The red particles denote the shell particles. The proper size particle (particle 3) is selected by settling down process while particles 1 and 2 flow aside. (**e**) Development of roughness of surface $\Gamma (t)$.

After starting the flow, local configuration of packing close to sidewall will self-organize into ordered state. The participating particles come from the boundary particles of the initial packing (see in Fig. 2a, Figure S3). Each particle in ordered state touches six neighboring wall particles to form a crystallized shell. This stable crystallized configuration initially appears at the bottom and then grows upwards. It takes several seconds for the crystallized shell to spread and reach the descending level. Usually, this shell is divided into some “mono-crystalline” cells with boundaries between them^10^, which are shown in Fig. 2c (also shown in Video 1). Different from the perfectly periodic chiral packing in small cylinder^33,34^, the boundaries appear because of the large size of the hopper and the fluctuations are induced during the formation of the shell. We mapped the shell particles to a 2D packing and surface packing density is calculated with being scaled by a hexagonal packing (0.907 for 2D disks)^33^, which is 0.99 for timepoint 12 s of Fig. 2c. This value indicates the shell is not, but close to a perfect hexagonal cell.

Large *μ*_pp_ will prevent particles moving to the most stable position (to form the hexagonal packing) and the weaken the whole structure. For contrast, small *μ*_pp_ helps to generate more wall particles to enforce the growth of the crystallized shell. Besides friction, as mentioned above, dispersity is known as another important factor for shear-induced ordering^8,35^. Our simulations also show that the distribution of particle size does influence the formation of the crystallized shell. The shell does not form when the global dispersity *λ* is large (0.1 for instance). A small *λ* guarantees the mechanical stabilization of the shell after the drainage. Figure 2b shows the size distribution in the crystallized shell. The dispersity in the shell is less than the global dispersity, which reveals segregation taking place during the drainage.

The shell growth is schematically illustrated in Fig. 2d. Already existing shell particles can be considered as the substrate during a crystal growth process. A boundary particle will become a wall particle with a probability of being pushed to touch the wall. Wall particles flow downwards with a motion involving sliding and rotation^36^ in contact with the wall. This process depends on the local configurations of the flowing region just above the top of the shell. Height of this region is about 2-3*d*_0_ as the motions of wall particles will be affected by inner particles. Main ingredient of this selection process is that the shell ‘prefers’ to choose particle that has about the same size (see in Fig. 2b), which leads to the narrower dispersity in the shell particles. This follows from the fact, that a narrower size dispersity helps to mechanically stabilize the shell.

We calculate roughness of this crystallized shell^37^, which is defined as $\Gamma (t)={(\frac{1}{2\mathrm{\pi }}{\int }_{0}^{2\mathrm{\pi }}{\left(h(\theta ,t)-\bar{h}(t)\right)}^{2}\mathrm{d}\theta )}^{1/2}$ here. The results are averaged from 20 parallel simulations and show the variation of *Γ* has an obvious relationship with time, which can be described with a power law of *t*: $\Gamma (t)\sim {t}^{\delta }$. It is surprising to find that the power *δ* = 1 (Fig. 2e). This result diverges from the predictions of both the Edwards-Wilkinson theory (*δ* = 1/2)^38^ and the KPZ theory (*δ* = 1/3)^39,40^.

### Growth rate

**Figure 3:**
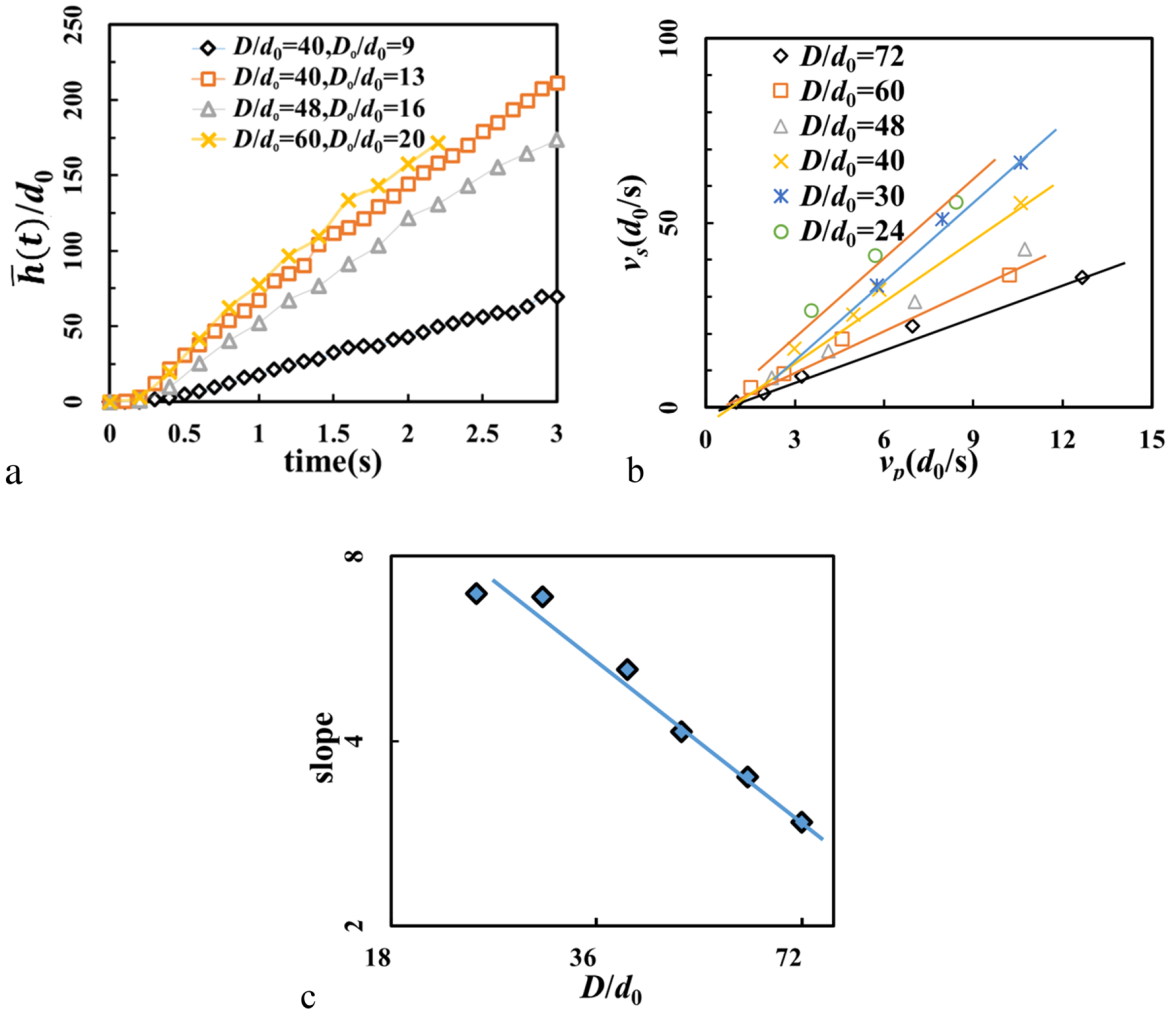
(**a**) Development of averaged shell surface height ($\bar{h}(t)$) with different *D* and *D*_0_. (**b**) The relation between *v*_*p*_ and *v*_*s*_. (**c**) Log–log plot of the slope of the linear relationship presented in Fig. 4b. Here *λ* = 0.

For every snapshot, averaged surface height of shell $\bar{h}(t)$ is calculated (see Fig. 3a). We see, that growth of the averaged surface height is nearly linear except at initial stage when the base of the shell is forming. During that stage there is no selection and only rearrangement is permitted, since particles near the bottom are stagnant. The slope of the linear region is defined as growth rate of the shell *v*_*s*_. The growth rate *v*_*s*_ with varying *μ*_pp_ and *μ*_pw_ are shown in Table 1. It drops from *v*_*s*_ = 76.0 *d*_0_/s when *μ*_pp_ = 0 to 12.6 as *μ*_pp_ grows to 0.1. The crystallized shell is no longer observed if *μ*_pp_ exceeds 0.1. It is also found that friction between particles and sidewall *μ*_pw_ has a minor effect to the formation of the shell. The influences of particle density, initial packing height and gravity to shell growth rate are shown in Table 2. Initial height and particle density have little influence while gravity will cause large difference via changing the flow rate.

**Table 1:** Growth rate of the shell *v*_*s*_.

*μ*_pw_	0.0	0.05	0.1	0.2	0.5	0.1			
*μ*_pp_	0.05					0.0	0.01	0.05	0.1
Grow rate *v*_*s*_ (*d*_0_/s)	26.3	26.2	25.9	24.5	22.9	76.0	58.8	25.9	12.6

Here *λ* = 0.

**Table 2:** Growth rate of the shell *v*_*s*_, varying with gravity, particle density and initial height.

*g* (m/s^2^)	0.981	9.81	98.1	9.81			9.81		
*ρ* (kg/m^3^)	2500.0			250.0	2500.0	25,000.0	2500.0		
Initial height *H*_0_ (*d*_0_)	625			625			375	625	1250
Grow rate *v*_*s*_ (*d*_0_/s)	10.3	25.9	72.9	22.2	25.9	29.6	25.5	25.9	26.1

*λ* = 0.

In analogy to the model of crystal growth^38^, it is natural to associate the growth rate to the particle velocity. Since the flowing particles above the surface of the shell have nearly the same velocity, velocity of potential shell particles can be calculated as: ${v}_{p}\approx \frac{\varphi }{{\rho }_{b}A}$, where $\varphi$ flow rate, ${\rho }_{b}$ bulk density of hopper flow and $A=\pi {D}^{2}/4$ is cross area of the hopper. We found that for each hopper size, there is a linear relationship between *v*_*s*_ and *v*_*p*_ (Fig. 3b). With small hopper size *D*, the slope of this dependence seems to have a limit value of 7. When $D\ge 30{d}_{0}$, the slope ~ *D*^-1^ (Fig. 3c). It is not checked here if the slope will drop to zero when *D* further increases.

### Shear layers

Our simulations show that growth of the crystallized shell is dominated by two parameters, the sliding friction coefficient *μ*_pp_ and the global dispersity of particles *λ*. In this section, three cases with different *μ*_pp_ and *λ* are presented for comparison while *μ*_pw_ is fixed to be 0.1 (see Table 3): case A: *μ*_pp_ = 0.05 and *λ* = 0; case B: *μ*_pp_ = 0.5 and *λ* = 0, and case C: *μ*_pp_ = 0.05 and *λ* = 0.1. We see crystallized shell only in case A, but not in cases B and C.

**Table 3:** Parameters in 6 cases.

Cases	A	B	C	A*	B*	C*
*μ*_pp_	0.05	0.5	0.05	0.05	0.5	0.05
*μ*_pw_	0.1	0.1	0.1	0	0	0
*λ*	0	0	0.1	0	0	0.1

**Figure 4:**
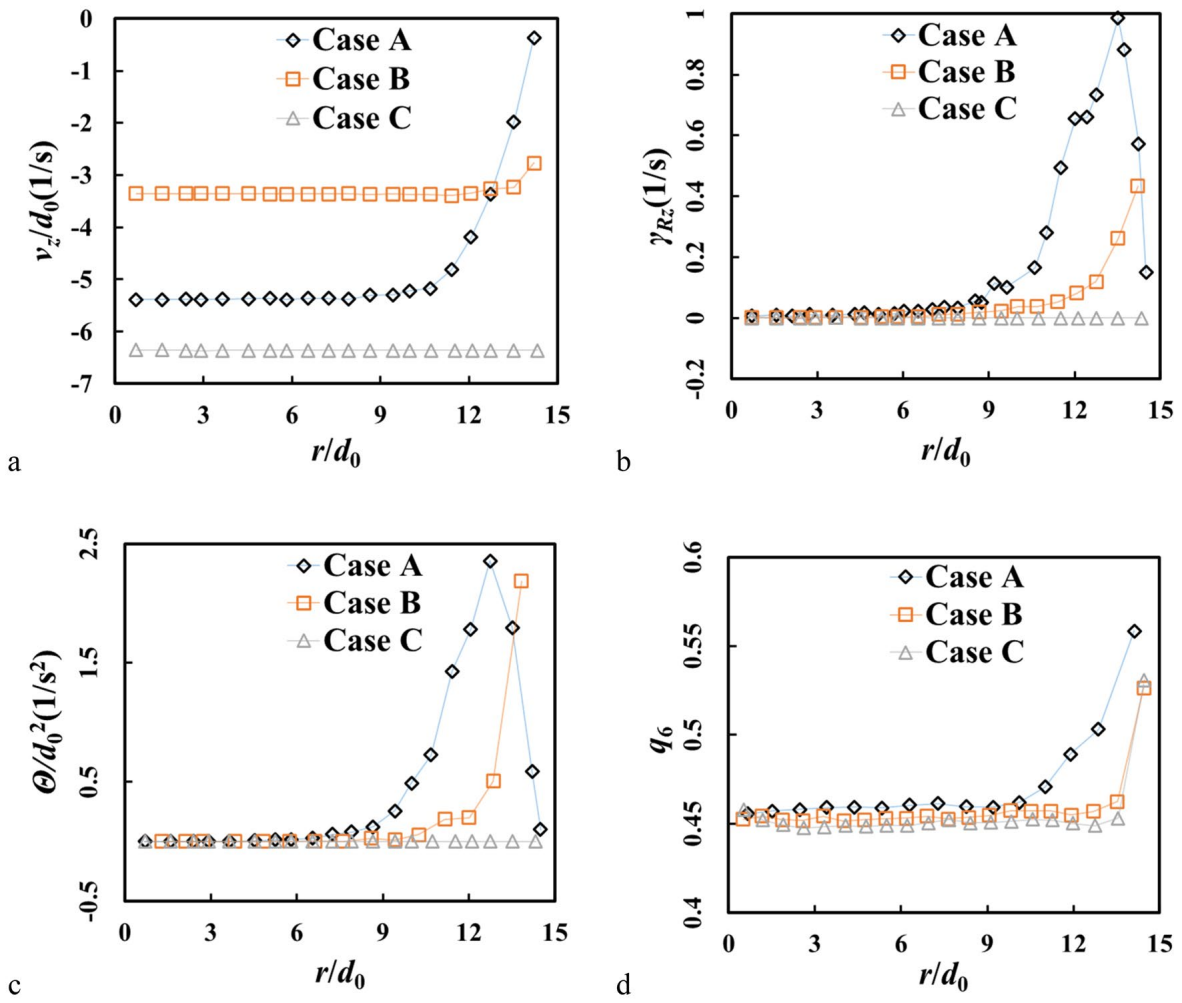
Radial profiles of (**a**) vertical velocity *v*_z_, (**b**) shear rate, (**c**) granular temperature, (**d**) orientational order *q*_6_ at *z* = 100 *d*_0_.

In case A, the wall particles are rearranged into hexagonal ordered state after flow begins. Value of local orientational order *q*_6_ is above 0.55, which is higher than that in the central region (0.45 is a typical value for disordered packing^41^) (see Fig. 4). Compared to the cases without crystallized shell (case B and case C), shear rate in the shear layers is obviously higher in case A. Due to the shear, granular temperature for particles in the shear layers is higher than in the central region, which is also the case seen in inclined flow^42^. The volume fraction close to the sidewall is slightly smaller in case B, which is consistent with local high temperature^43^. When *λ* is 0.1 (case C), a plug-like flow occurs and *v*_z_ is much larger than in cases A and B. Both the shear rate and the granular temperature are then close to zero.

**Figure 5:**
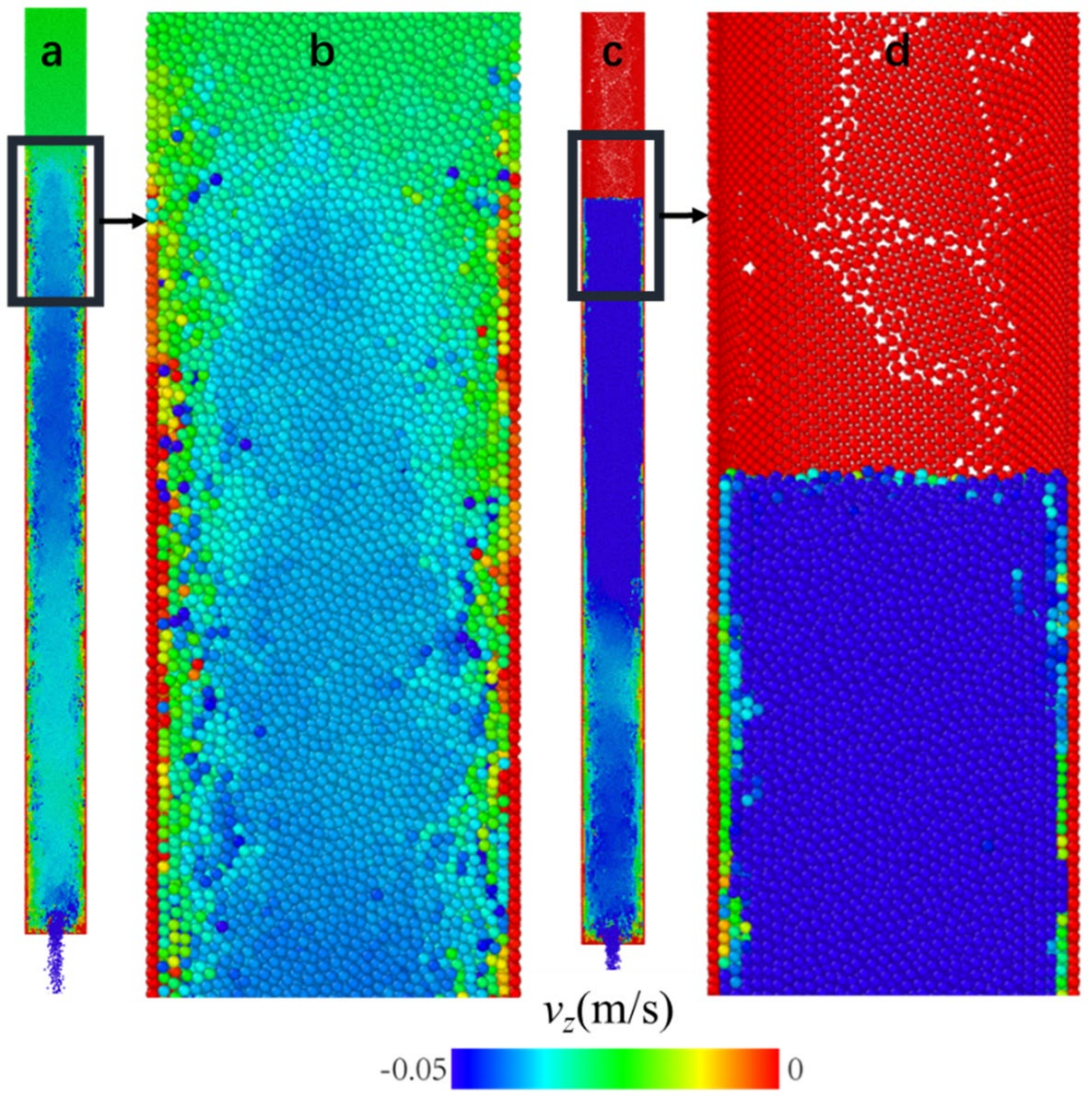
(**a**) A cross section of the flow at a stage when the crystallized shell does not reach the descending top surface. (**b**) Enlarged picture of the part around the growing shell surface in (**a**). (**c**) The cross section of the flow when the top surface level is below the shell surface. (**d**) Enlarged picture of the part around the top surface in (c), with the developed shell reaching well above the surface. The results in this figure is visualized by^45^.

It is interesting that during the growth of the crystallized shell, there are two coexisting phases of the flow (see Fig. 5). In the area where the crystallized shell has formed, the ordered wall particles are nearly static, and shear is concentrated near the shell. Above this area where crystallized shell is not developed yet, *v*_*z*_ of all particles is still nearly uniform and no obvious shear layer is observed. Thus, the occurrence of shear layers is due to this crystallization. The shear flow region expands upwards until the growing crystallized shell encounters the top surface of the descending level. In our simulation, funnel flow is not observed even if *μ*_*pp*_ reaches 0.5, perhaps because wall friction is not enough here^44^. The crystallized shell remains static after the drainage, which is found in our experiment with spherical glass particles and steel particles (see Figure S2 and Video 2). Moreover, the growth rate is measured and the value is 7.42 ± 0.53 *d*_0_/s, which is less than that in simulations.

**Figure 6:**
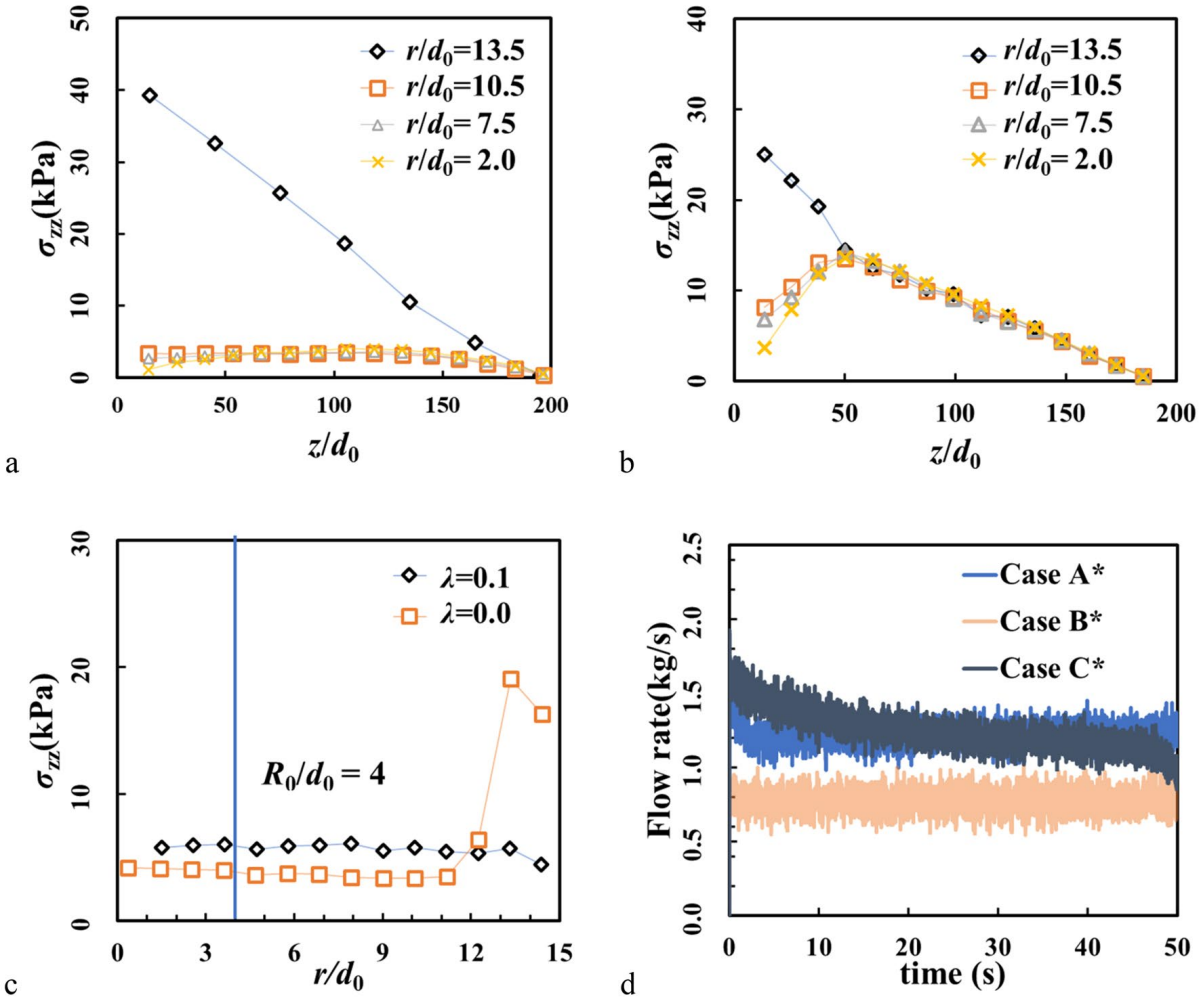
Vertical stress *σ*_zz_ along *z* axis in (**a**) Case A* (*λ* = 0) and (**b**) in Case C* (*λ* = 0.1) (see Table 3). (**c**) *σ*_zz_ in the radial direction at *z*/*d*_0_ = 100. The vertical line marks the radius of outlet. (**d**) Temporal profile of instantaneous mass flow rate in Cases A*, B* and C*.

Compared with the reported crystallizations mentioned above^7,9,10^, the phenomenon found in our case is however different. First of all, the sidewall of hopper in our case is vertical and smooth. Mechanical stability of the crystallized shell is supported by the bottom and where the shell is formed, it is like a frictional wall, leading to strong shear in the neighboring layers. Therefore as shown in Fig. 6a, a Janssen-like stress profile still exists in a frictionless hopper, similar to the one observed in the normal frictional hoppers^46,47^. As the weight of particles is supported by the shell, there is a strong peak of vertical stress at the boundary (see in Fig. 6c). For comparison, when *λ* = 0.1, *σ*_*zz*_ behaves like hydrostatic pressure, which is linear with height except near the bottom (Fig. 6b).

Variations of instantaneous flow rates with time are shown in Fig. 6d. In previous DEM simulations, flow rate will decrease with time by inducing a small wall friction^46,48^. Interestingly, our results show that flow rate is constant with the growing crystallized shell (Case A*) and decreases in the situation without the shell (Case C*), which questions the relationship between the flow rate and the roughness (or friction) of sidewalls. The flow rate in Case B* is smaller than that in both of Cases A* and C*, which verifies the influence of particle–particle friction reported in previous studies^22,23^.

## Conclusion

We have investigated numerically of the experimental observation that a layer of crystallized shell formed at the container wall during hopper flow. Our simulation shows degree of the particle polydispersity and particle–particle friction are two main factors to prevent the shell formation. Those particles being pushed to the wall by the flowing particles, tend to pile up from the base and grow to the top during the flow. The faster the flow rate is, the faster the growth rate of the shell layer piles. The ratio of the rates depends on the container diameter. The smaller the container the larger the ratio is. Once it is formed, the shell remains there, even when the flow ends. This formed shell serves as a new wall, which guarantees the flow rate constant no matter what wall material is. This phenomenon is interesting not only for its formation of crystallized shell with no need of external drive, but also for its altering the flow properties and stabilizing the flow rate. More simulations and experiments should be done to check this phenomenon in non-spherical particle system in the future.

## Methods

### Simulation method

To model the dynamical behavior of particles, our simulations are carried out on multiple GPUs using the DEM code (Discrete Element Method) developed by us^49^. DEM is a widely used simulation tool in granular mechanics, in which particle positions, velocities and interactions are tracked by equations of motion, simple contact models being then provided. In our simulation, the soft-particle contact model is used where the interactions consisting of elastic and viscous components in normal and tangential directions are calculated from the overlap and its time rate of change. By the Hertz-Mindlin contact model^50,51^, the normal and tangential contact forces between two contacting particles are:

2 $$\left\{\begin{array}{c}{\boldsymbol{F}}_{i{j}_{n}}=\frac{4}{3}{Y}_{\text{eff}}\sqrt{{R}_{\text{eff}}{\delta }_{i{j}_{n}}}{\boldsymbol{\delta }}_{i{j}_{n}}-2\sqrt{\frac{5}{6}}\beta \sqrt{2{Y}_{\text{eff}}\sqrt{{R}_{\text{eff}}{\delta }_{i{j}_{n}}}{m}_{\text{eff}}}{\boldsymbol{v}}_{i{j}_{n}}\\ {\boldsymbol{F}}_{i{j}_{t}}=8{G}_{\text{eff}}\sqrt{{R}_{\text{eff}}{\delta }_{i{j}_{n}}}{\boldsymbol{\delta }}_{i{j}_{t}}-2\sqrt{\frac{5}{6}}\beta \sqrt{8{G}_{\text{eff}}\sqrt{{R}_{\text{eff}}{\delta }_{i{j}_{n}}}{m}_{\text{eff}}}{\boldsymbol{v}}_{i{j}_{t}}\end{array}.\right.$$

3 $$ \beta {\text{ = }}\frac{{\ln e}}{{\sqrt {\ln ^{2} e + \pi ^{2} } }}. $$

4 $${Y}_{\text{eff}}=1/((1-{\nu }_{i}^{2})/{Y}_{i}+(1-{\nu }_{j}^{2})/{Y}_{j}).$$

5 $${G}_{\text{eff}}=1/(2(2+{\nu }_{i})(1-{\nu }_{i})/{Y}_{i}+2(2+{\nu }_{j})(1-{\nu }_{j})/{Y}_{j}).$$

6 $${r}_{\text{eff}}={r}_{i}{r}_{j}/({r}_{i}+{r}_{j}).$$

7 $${m}_{\text{eff}}={m}_{i}{m}_{j}/({m}_{i}+{m}_{j}).$$

where *G* is the shear modulus, *Y* is the Young’s Modulus, *r* is the radius of the particles, *m* is the mass of particles and *e* is the coefficient of restitution between particles. **δ**_*ij*n_ and **δ**_*ij*t_ are normal and tangential displacement vectors, and *δ*_*ij*n_ and *δ*_*ij*t_ are their modules, respectively. **v**_*ij*n_ and **v**_*ij*t_ are normal and tangential relative velocities between the particles *i* and *j*. **δ**_*ij*n_ ≡ (*R*_*i*_ + *R*_*j*_ – *r*_*ij*_)**r**_*ij*_/*r*_*ij*_ and *δ*_*ij*t_ is determined by integrating **v**_*ij*t_. Considering sliding friction, the Coulomb yield criterion F_*ij*t_ ≤ *μ*_s_F_*ij*n_ is satisfied by truncating tangential overlap **u**’_*ij*_ = *μ*_s_F_*ij*n_**u**_*ij*_/F_*ij*t_^51^. *μ*_s_ is the sliding friction coefficient between particles. **u**’_*ij*_ and **u**_*ij*_ are truncated and original tangential overlap respectively_._ Further more, rolling and torsion friction should be introduced for irregular shaped particles. These influences are given by rolling and torsion torques:

8 $$\left\{\begin{array}{c}{\boldsymbol{M}}_{r}=-{\mu }_{r}{F}_{i{j}_{n}}{\widehat{\boldsymbol{\omega }}}_{ij}\\ {\boldsymbol{M}}_{t}=-{\mu }_{t}{F}_{i{j}_{n}}{\widehat{\boldsymbol{\omega }}}_{ij}\end{array}\right..$$

*μ*_r_ and *μ*_t_ are rolling and torsion frictions respectively, ${\widehat{\mathbf{\omega }}}_{ij}$ is the relative angular velocity between two contacting particles.

Under gravity field, the equations of motion of the particles are:

9 $$\left\{\begin{array}{c}{m}_{i}{\boldsymbol{a}}_{i}={\sum }_{j}\left({\boldsymbol{F}}_{i{j}_{n}}+{\boldsymbol{F}}_{i{j}_{t}}\right)+{m}_{i}\boldsymbol{g}\\ {I}_{i}{\dot{\boldsymbol{\omega }}}_{i}={\sum }_{j}\left[-\frac{{r}_{i}}{{r}_{ij}}{\boldsymbol{r}}_{ij}\times \left({\boldsymbol{F}}_{i{j}_{n}}+{\boldsymbol{F}}_{i{j}_{t}}\right)\right]\end{array}\right..$$

These equations are solved by integration using the Velocity-Verlet scheme^52^. The model parameters used in our simulations are listed in Table 4.

**Table 4:** Case parameters in simulations.

Physical quantity	Symbol	Value
Averaged particle diameter	*d*_0_	6.0 mm
Global dispersity	*λ*	0.0, 0.05, 0.1
Particle density	*ρ*	2850 kg/m^3^
Elastic modulus	*Y*	72 GPa
Poisson’s ratio	*ν*	0.25
Particle–particle friction coefficient	*μ*_pp_	0, 0.01, 0.05, 0.1, 0.5
Particle–wall friction coefficient	*μ*_pw_	0, 0.05, 0.1, 0.2, 0.5
Particle–particle and particle–wall coefficient of restitution	*e*	0.6
Particle–particle and particle–wall rolling friction coefficient	*μ*_*r*_	1 mm
Particle–particle and particle–wall torsion friction coefficient	*μ*_*t*_	0.4 mm
Outlet diameter	*D*_0_	8 *d*_0_
Hopper diameter	*D*	30 *d*_0_

In our simulations, dispersed glass spheres were randomly generated with a small volume fraction within flat bottomed hoppers and then they were packed under gravity till the total kinetic energy of the packing was small enough (< 10^–10^ J). During the packing process, a viscous damping was added to accelerate the packing process and make a reasonable stress distribution in conformity with the Janssen’s effect^53^. The volume fraction of the packing is roughly 0.60. After the packing process, the orifices of hoppers were opened to let the particles flow.

### Definitions of quantities

The size distribution of particles in the simulation is uniform in the rage ((1–1/2*λ*)*d*_0_, (1 + 1/2*λ*)*d*_0_), where *λ* is named to be the global dispersity of particles. The local orientational factor *q*_6_^54,55^ and the granular temperature^56^ are calculated in our system. The *q*_6_ of the *i*-th particle is given by:

10 $${q}_{6}={\left[\frac{4\pi }{13}{\sum }_{m=-6}^{6}{\left|{\bar{Q}}_{6m,i}\right|}^{2}\right]}^{1/2},$$

where ${\bar{Q}}_{6m,i}=\frac{1}{{N}_{i}}{\sum }_{j}^{i}{Q}_{6m}\left({\overrightarrow{r}}_{ij}\right)$, and ${\overrightarrow{r}}_{ij}$ is the midpoint between particle *i* and *j*, *Q*_6m_ is spherical harmonics *Y*_*lm*_ when *l* = 6. In our simulation, granular temperature in *z* direction is given by:

11 $$\Theta =\langle {v}_{z}^{2}\rangle-{\langle{v}_{z}\rangle}^{2}.$$

The bracket denotes an average value of particle velocities within a volume *Ω.* $\langle{v}_{z}\rangle$ is the corresponding local average vertical velocity. The stress is calculated by virial stress^57^:

12 $$ \sigma _{{\alpha \beta }}  = \frac{1}{\Omega }\sum\limits_{{i\;in\;\Omega }} {\left( { - m^{i} \left( {v_{\alpha }^{i}  - \overline{{v_{\alpha } }} } \right)\left( {v_{\beta }^{i}  - \overline{{v_{\beta } }} } \right) + \frac{1}{2}\sum\limits_{j} {\left( {x_{\alpha }^{j}  - x_{\alpha }^{i} } \right)f_{\beta }^{{ij}} } } \right)}  $$

where ${m}^{i}$ is the mass of the *i*th particle in a volume $\Omega$ ,${x}_{\alpha }^{j}$ its position with Cartesian components, ${v}_{\alpha }^{i}$ its velocity, $\bar{{v}_{\alpha }}$ the local average velocity, and ${f}_{\alpha }^{ij}$ is the force on molecule exerted by another particle.

The shear rate is given by:

13 $${\Gamma }_{\alpha \beta }=\mathrm{\Delta }\langle{v}_{\alpha }\rangle/\mathrm{\Delta }{L}_{\beta }$$

$\langle{v}_{\alpha }\rangle$ is the average velocity of particles in a cell along $\alpha$ direction, and $\mathrm{\Delta }{L}_{\beta }$ is the cell size along the $\beta$ direction.

### Experiment setup

In the experiment (shown in Figure S1), spherical glass particles and steel particles are used with the diameter of 6 ± 0.02 mm. The cylindrical hoppers with flat bottom are made of two materials: transparent plexiglass and steel. Both diameters of the hoppers are 100 mm and the openings at the bottom center have a diameter of 32 mm. The experimental facility is fixed on a damping platform. The environment temperature is 21 °C with a relative humidity is 35%.

Before the experiments, the hopper is set upright vertically with blocked opening, and particles are filled up to the height of 1500 mm. A high-speed camera is fixed on a tripod to shoot videos of the particles close to the wall at a frame rate of 100 fps.

## Supplementary Information

Supplementary Video 1.

Supplementary Video 2.

Supplementary Video 3.

Supplementary Information.
