# Supplementary material for: Flow-induced surface crystallization of granular particles in cylindrical confinement
Source: Sci Rep. 2021 Jun 24;11:13227. doi: 10.1038/s41598-021-92136-9 (PMC8225843; doi:10.1038/s41598-021-92136-9)
Supplement: Supplementary file 4 — Supplementary Information. [file 41598_2021_92136_MOESM4_ESM.docx]

**Shell crystallization in granular hopper flow**

Sheng Zhang^1,2,^*, Ping Lin^1,2,^*, Mengke Wang^1,2^, Jiang-feng Wan^3^, Yi Peng^1,2^, Lei Yang^1,2,4,✉^_,_ Meiying Hou^2,5,✉^

^1^ Institute of Modern Physics, Chinese Academy of Sciences, Lanzhou, 730000, China

^2^ University of Chinese Academy of Sciences, Beijing, 100049, China

^3^ East China University of Technology, Nanchang, 330105, China

^4^ Lanzhou University, Lanzhou, 730000, China

^5^ Institute of Physics, Chinese Academy of Sciences, Beijing, 100190, China

*Zhang S and Lin P contributed equally to this work.

^✉^Correspondence and requests for materials should be addressed to Hou M (email: mayhou@iphy.ac.cn) or to Yang L (email: [lyang_imp@outlook.com](mailto:lyang_imp@outlook.com))

### Supplemental figures


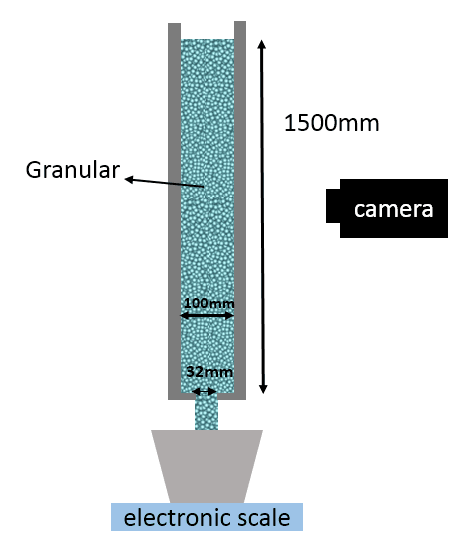


particles

Figure S1 The experiment setup.


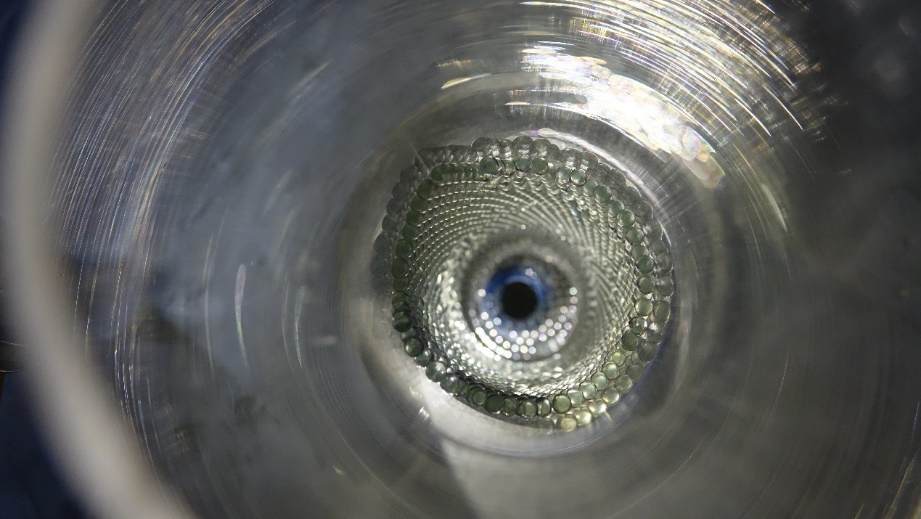


**a**

**b**


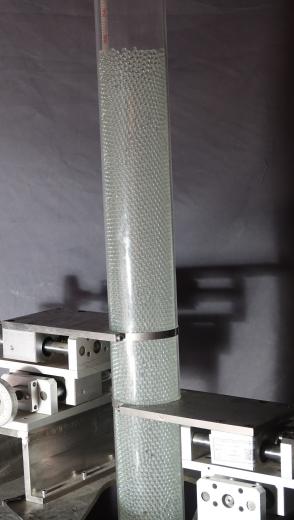


Figure S2 The remaining crystallization shell (glass particles in a transparent plexiglass tube). a) Side view. b) Top view. Noted there is a reflection image of the plexiglass tube.


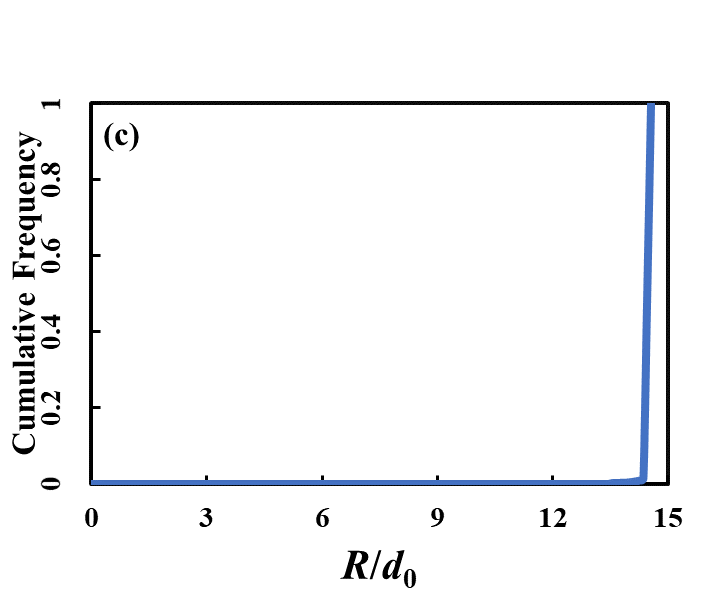


Figure S3 Cumulative frequency of initial radial distribution of the final shell particles. This result shows nearly all shell particles come from the initial boundary particles.

### Simulation of Fluids

The rate of flow of the viscous Newtonian fluid discharging from a 2D hopper was simulated by CFD codes. The hopper (shown in Figure S4) is 300mm-high and the diameter is 400mm with flat bottom and 15mm-wide opening in the center (out 2 mm). Initially the level of fluid is 250 mm high (density *ρ_f_*=1400 kg m^-3^, varying viscosity *η_f_*=3, 8, 10, 30, 100 Pa•s) and environment gas is air (*ρ_a_*=1.225kg m^-3^, *η_a_*=1.7894×10^-5^ Pa•s). The viscous model is standard K-Epsilon and the standard wall function is used near the wall. A no-slip boundary condition is imposed at the wall and a zero-pressure condition is imposed at the level and at the outlet.

To simulate a Bingham fluid, by following Staron et al. [1], we set $=min(\frac{\mu\boldsymbol{P}}{\boldsymbol{D}_{2}},\eta_{max})$ , where *μ* is the effective coefficient of friction of the granular flow: $\mu=\mu_{s}+\frac{\Delta\mu}{1+\frac{I_{0}}{I}}$, where $\mu_{s}$, $\Delta\mu$, and $I_{0}$ are constants. ***P*** is the local pressure and $D_{2}$ is the second invariant of the strain rate tensor ***D***: $\boldsymbol{D}_{2}=\sqrt{\boldsymbol{D}_{ij}\boldsymbol{D}_{ij}}$. *I* is a dimensionless parameter: $I={d\boldsymbol{D}_{2}}/{\sqrt{\boldsymbol{P}/\rho}}$, where *ρ* is the density of the spheres. In the same way as for the simulation of a Newtonian fluid, the system is simplified to a 2D one, and the standard K-Epsilon model is employed. A no-slip boundary condition is imposed at the wall near which the standard wall functions is used.

In the simulation of drainage of viscous Newtonian fluid from a cylindrical hopper, a decreasing flow rate and the clinging of a thin layer to the wall are observed. Similar behavior of honey is observed in the experiment and the viscosity is about 8$Pa\cdot s$. For different height, there is a uniform law of the variations of thicknesses of the layer with heights (see Figure S6). This variation can be described by fitting of a power-law formula of time *t*: $l\left( t \right)=a\cdot t^{b}$, here *a*=0.006, the value of *b* is -0.511 which is slightly smaller than -1/2 in [2]. The layer becomes thinner and thinner and breaks into drops which will slide down the wall very slowly.

In [1], the constant flow rate and pressure cavity will be successfully reproduced by simulating a Bingham fluid and employing *μ*(*I*) rheology. We follow this work to find if there exists a permanent existing boundary layer. The results show unlike the viscous Newtonian fluid, there is no boundary layer and shear layer. The flow pattern is plug-like and similar with DEM results with large *μ*_pp_ (>0.1) (Figure 4).


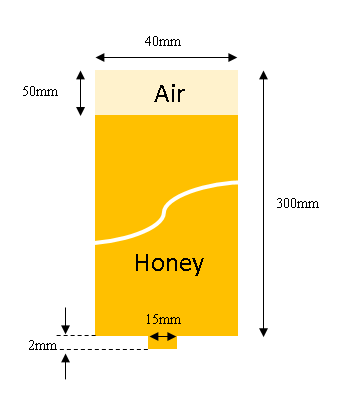


Figure S4 Simulation of honey flow.


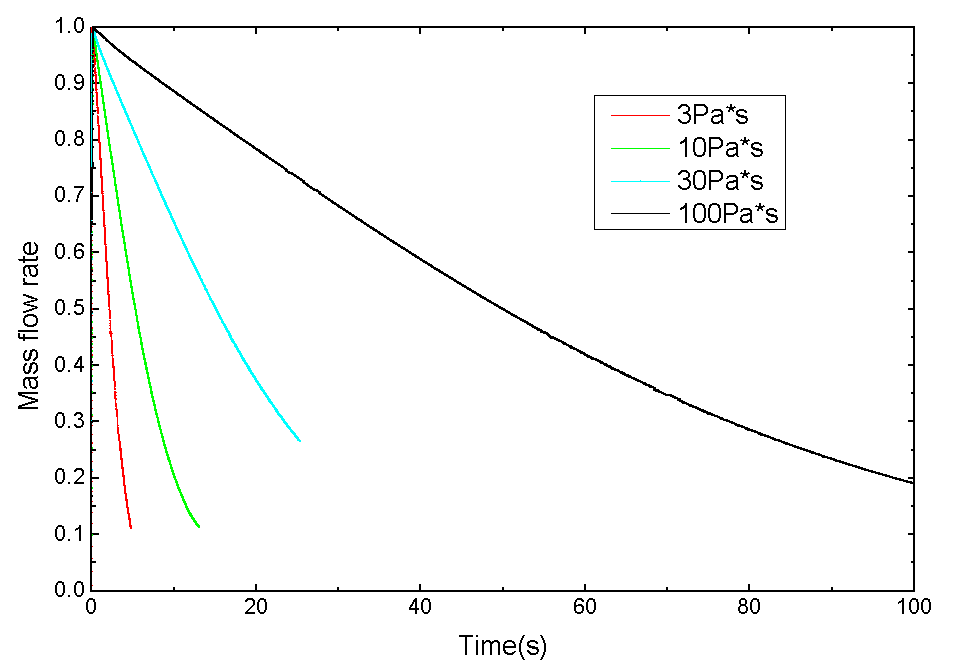


Figure S5 Drainage of viscous Newtonian fluid with a range of viscosity.


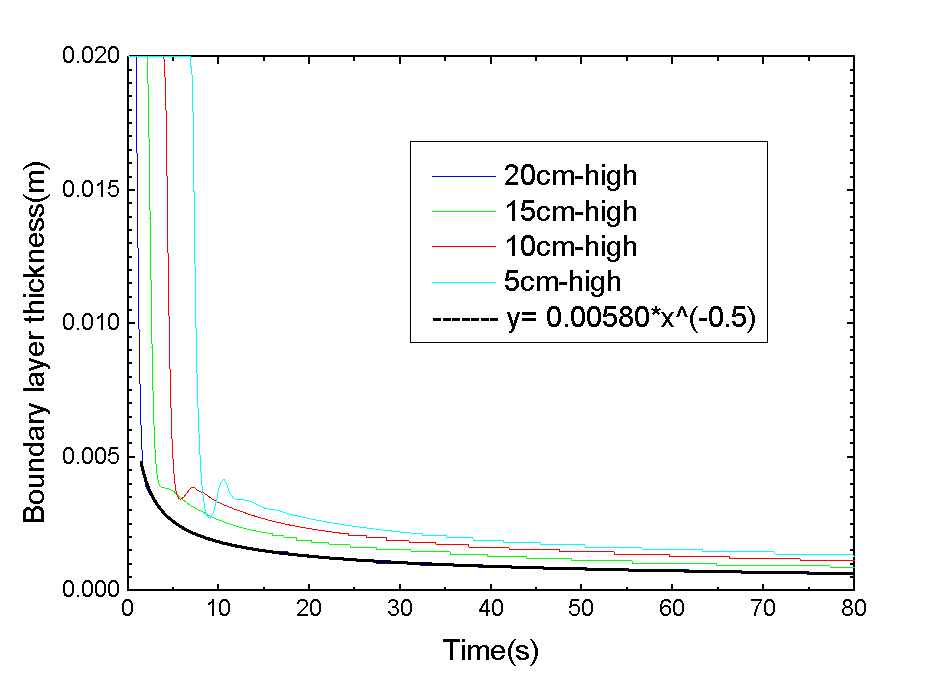


Figure S6 Variation of thickness of boundary film at different heights.

### Videos

Video 1 Experiment results using spherical glass particles and hopper with transparent plexiglass

Video 2 Experiment results using spherical steel particles and steel hopper

Video 3 A movie of simulation results

### References

1. Staron, L., P.Y. Lagree, and S. Popinet, *The granular silo as a continuum plastic flow: The hour-glass vs the clepsydra.* Physics of Fluids, 2012. **24**(10).

2. Jeffreys, H., *The draining of a vertical plate.* Proceedings of the Cambridge Philosophical Society, 1930. **26**: p. 204-205.
